# Quality characteristics, lysozyme activity, and albumen viscosity of fresh hatching duck eggs after a week's storage at various temperatures

**DOI:** 10.1038/s41598-024-56351-4

**Published:** 2024-03-07

**Authors:** Sebastian Wlaźlak, Zuzanna Brzycka, Weronika Ragus, Mirosław Banaszak, Małgorzata Grabowicz

**Affiliations:** Department of Animal Breeding and Nutrition, Faculty of Animal Breeding and Biology, Bydgoszcz University of Science and Technology, Mazowiecka 28, 85-084 Bydgoszcz, Poland

**Keywords:** Biochemistry, Zoology

## Abstract

The study aimed to analyze the qualitative features of Cherry Valley duck’ hatching eggs during storage at different temperatures. Eggs were divided into 3 equal groups with 30 eggs each: fresh egg and stored at 7 °C and 17 °C within one week. Qualitative analyses of duck eggs were carried out, considering the morphological composition, physicochemical characteristics, lysozyme activity, and albumen viscosity. The highest weight of yolk and its percentage was found in the 17 °C group. The weight and percentage of albumen were significantly the highest in the group of fresh eggs. Higher egg weight loss was observed in the group stored at higher temperatures. Higher thick albumen height and Haugh units were found in fresh eggs and eggs stored at 7 °C. Different temperatures of egg storage did not affect lysozyme activity in thick and thin albumen. Stored eggs were characterized by lower albumen viscosity only at a shear rate of 10 rpm. The higher viscosity of thick albumen compared to thin ones was demonstrated at 10 and 20 rpm shear rates. The presented research results indicate a large diversity of selected qualitative indicators of hatching duck eggs, which may affect their storage and suitability for incubation.

## Introduction

A high percentage of hatched ducklings is one of the leading indicators determining poultry hatching quality. It is also influenced by genetic factors, nutrition, diseases, egg quality, flock age, egg storage, and incubation conditions^1,2^. Egg storage conditions (temperature, humidity) mainly determine their quality changes and correspond to hatchability and chick quality. It is also recommended that hatching eggs should not be stored for more than 3–5 days. Extending this time (over day 7) has a negative impact on, among others, incubation time, mortality, and chick quality^3^. At a temperature below 24 °C, the metabolic processes of the avian embryo are entirely inhibited. It is defined as physiological zero^4^.

Environmental conditions during the storage of duck eggs determine the rate of quality changes, which affects their biological value for hatching^5^, similarly to the duration of the storage period^6^. Dassidi et al.^7^ analyzed the influence of egg storage length on the quality and hatchability of Cherry Valley ducklings. After 14 days of storage, the authors found a significant decrease in Haugh units (HU) and albumen weight, which resulted in poorer viability and hatchability of the chicks. According to Pereira et al.^8^, after 10 days of Pekin duck egg storage, significant changes in the albumen quality were observed, negatively affecting the quality of hatchability. It was suggested that duck egg storage for less than 7 days allows for the highest hatchability of ducklings^9^. The size of duck eggs influences the rate at which changes occur when stored at room temperature. The best parameters were characterized by eggs of medium weight (63.2–73.6 g), confirmed in the study with Magelang duck eggs^10^. The cited authors also indicated a significant decrease in egg quality parameters after just 3 days of storage. This suggests the importance of the genetic factor in the rate of quality changes during egg storage^5^. The studies also pointed to the age of the flock, which may determine changes in the quality of eggs during storage^11^. According to Quan and Benjakul^12^, several changes in duck eggs during storage were found, including egg weight loss, CO_2_ release, thinning of thick albumen, increase in albumen pH, decrease in trypsin inhibitors, and increase in S-ovalbumin. The authors also observed a decrease in the foaming properties of the albumen, which are usually much higher in duck egg albumen than in chicken ones.

Previous studies have shown the significant importance of temperature and storage time in changes in selected egg characteristics. Changes in egg weight, HU, or air cell size (one of the leading indicators of egg freshness) were found^13^. As demonstrated in the research of Feddern et al.^14^, storage of eggs at room temperature resulted in a decrease in the height of the thick albumen and yolk, HU, and an increase in the pH of the yolk in a much shorter time. The lower temperature was conducive to inhibiting changes leading to egg quality deterioration. Notably, relationships have been shown in a study where the Bovans White laying hen eggs were stored at 5, 21, and 29 °C. Higher temperatures significantly reduced the height of the thick albumen, HU, and increased the pH of the albumen and the air cell size, which is associated with more intensive water evaporation and egg weight loss^15^. Similar conclusions were also presented by Martinez et al.^16^ and Carvalho et al.^17^.

Lysozyme is one of the main proteins in eggs with high antibacterial properties. It is a protective barrier, specifically against Gram-positive bacteria. The so-called lysozyme activity measures the antimicrobial effectiveness of this protein as protection of the egg or the embryo against the penetration of pathogenic microorganisms^18^. The study of Schäfer et al.^19^ showed that neither storage time nor temperature affected lysozyme activity in chicken eggs. In addition, it was recommended to use a temperature range of 5–8 °C due to better hygienic conditions and less impact on the deterioration of egg quality than the use of higher temperatures. However, lysozyme activity may decrease during the storage of eggs^20,21^. Albumen viscosity is another parameter for evaluating egg quality due to its association with its ability to foam and emulsify. The rheological properties of the albumen are characterized by high variability due to the type of albumen (thin or thick) and the method of preparing samples for analysis^22^.

The available literature on the quality assessment of hatching duck eggs is limited. The selected issues presented in the research (lysozyme activity, albumen viscosity) have often been analyzed in more detail in eggs of other poultry species. Cherry Valley ducks (SM3 European Medium) are the most common hybrid of meat-type ducks in the world^23^; therefore, this study focused on the analysis of the quality of eggs of duck origin following current market trends and may provide support in the aspect of storage, including the hatchability of ducklings. The study aimed to assess the impact of fresh hatching duck eggs after one week of storage at different temperatures (7 °C and 17 °C) on the morphological and physicochemical features that define their biological value.

## Results

It was seen that the storage at different temperatures affected the egg weight. Notably, significantly lower egg weight was found in 17 °C than in 7 °C and fresh eggs. The eggs stored at 7 °C also reduced the weight, which was statistically confirmed due to the initial egg weight (P < 0.001). After storing the eggs at 17 °C, a significantly higher yolk weight was found compared to the group of fresh eggs (P = 0.005). A significantly higher percentage of yolk was found in both groups where the eggs were stored for 7 days. Fresh eggs were characterized by significantly higher weight and percentage of albumen concerning the remaining groups. In the case of eggshell weight, significant differences were found between fresh eggs and those stored at 17 °C (Table 1).

**Table 1:** Weight loss and morphological composition of fresh duck eggs or after one week of storage at different temperatures.

Item (n = 30)	Group^1^						SEM	P-value^2^
	*x̅* Fresh	± SD	*x̅* 17 °C	± SD	*x̅* 7 °C	± SD		
Egg								
Initial egg weight (g)	88.36	5.32	85.91	3.70	86.80	3.25	0.45	0.077
Weight loss (%)	0.00^c^	0.00	0.94^a^	0.30	0.51^b^	0.12	0.04	< 0.001
Width (mm)	48.30	2.31	48.29	0.97	48.64	0.76	0.16	0.598
Length (mm)	66.38	1.72	66.43	2.30	66.21	2.16	0.22	0.909
Shape index (%)	72.79	3.64	72.78	3.04	73.53	2.62	0.33	0.564
Weight (g)	88.36	5.32	85.91	3.70	86.80	3.25	0.45	0.077
Surface area (cm^2^)	93.90	3.69	92.18	2.63	92.82	2.29	0.31	0.077
Yolk								
Weight (g)	29.11^b^	2.68	31.35^a^	2.62	30.47^ab^	2.45	0.29	0.005
Share (%)	32.95^b^	2.34	36.53^a^	3.03	35.11^a^	2.58	0.32	< 0.001
Albumen								
Weight (g)	51.29^a^	3.79	46.96^b^	3.84	48.50^b^	2.89	0.41	< 0.001
Share (%)	58.05^a^	2.43	54.62^b^	3.16	55.87^b^	2.45	0.32	< 0.001
Eggshell								
Weight (g)	7.96^a^	0.68	7.59^b^	0.51	7.83^ab^	0.50	0.06	0.047
Share (%)	9.00	0.49	8.85	0.63	9.02	0.50	0.06	0.425

^1^Group: Fresh, fresh eggs; 17 °C, eggs stored at 17 °C for 7 days; 7 °C, eggs stored at 7 °C for 7 days.

^2a^^,b^Mean values with no common superscript differ statistically significantly, P < 0.05.

The darker yolks (DSM) were found in the group stored at 17 °C (P = 0.039). On the other hand, considering the instrumental analysis of the yolk color, this group also had the highest L* and b* values compared to the other groups. The lowest yolk pH was found in fresh eggs (P < 0.001). The fresh eggs and eggs stored at a lower temperature were characterized by significantly higher thick albumen and higher values of HU (P < 0.001). In contrast to the pH of thick and thin albumen, the lowest values were shown in these groups than in the 17 °C group (P < 0.001). The thickness of the eggshell was significantly different between all groups (Table 2).

**Table 2:** Physicochemical features of fresh duck eggs or after one week of storage at different temperatures.

Item (n = 30)	Group^1^						SEM	P-value^2^
	*x̅* Fresh	± SD	*x̅* 17 °C	± SD	*x̅* 7 °C	± SD		
Vitelline membrane strength (N)	0.13	0.03	0.13	0.02	0.13	0.02	0.00	0.491
DSM	5.20^b^	0.41	5.57^a^	0.68	5.50^ab^	0.63	0.06	0.039
L*	50.04^b^	1.93	51.74^a^	1.53	50.26^b^	1.89	0.20	0.001
a*	− 4.01	0.36	− 4.10	0.56	− 3.58	1.62	0.11	0.107
b*	33.79^b^	2.54	38.78^a^	1.99	34.95^b^	6.46	0.49	< 0.001
pH yolk	6.10^c^	0.05	6.28^a^	0.10	6.15^b^	0.03	0.01	< 0.001
Thick albumen height (mm)	6.90^a^	0.86	5.51^b^	1.05	6.63^a^	1.00	0.12	< 0.001
Haugh units	73.91^a^	7.40	60.87^b^	14.60	72.08^a^	7.50	1.24	< 0.001
pH thick albumen	9.04^b^	0.07	9.26^a^	0.16	9.04^b^	0.03	0.02	< 0.001
pH thin albumen	9.01^b^	0.14	9.23^a^	0.13	9.02^b^	0.09	0.02	< 0.001
Eggshell strength (N)	38.34	7.96	35.85	8.56	37.77	8.08	0.86	0.473
Strength/1 g eggshell (N)	4.80	0.81	4.69	1.04	4.82	0.96	0.10	0.839
Eggshell thickness (mm)	0.356^a^	0.024	0.330^b^	0.029	0.310^c^	0.031	0.004	< 0.001

L* lightness, a* redness, b* yellowness, *SEM* standard error of the mean.

^1^Group: Fresh, fresh eggs; 17 °C, eggs stored at 17 °C for 7 days; 7 °C, eggs stored at 7 °C for 7 days.

^2a^^,b^Mean values with no common superscript differ statistically significantly, P < 0.05.

Significantly higher lysozyme activity was found in thin albumen from fresh eggs than fresh thick albumen, as well as thick albumen stored at different temperatures for 7 days. At the same time, thin albumen stored for 7 days at 7 °C and 17 °C was characterized by significantly higher lysozyme hydrolytic activity compared to thick albumen after a week of storage (P < 0.001) (Table 3).

**Table 3:** Lysozyme activity in thick and thin albumen from fresh duck eggs or after one week of storage at different temperatures.

Group^1^	Lysozyme activity (U/ml)	± SD
Thin albumen		
*x̅* fresh	14,980.00^a^	2361.05
*x̅* 17 °C	13,526.67^ab^	3401.78
*x̅* 7 °C	12,293.33^ab^	4279.10
Thick albumen		
*x̅* fresh	11,007.14^bc^	3399.43
*x̅* 17 °C	8693.33^c^	1745.39
*x̅* 7 °C	7786.67^c^	2395.79
SEM	415.11	
P-value^2^	< 0.001	

^1^Group: Fresh, fresh eggs; 17 °C, eggs stored at 17 °C for 7 days; 7 °C, eggs stored at 7 °C for 7 days.

^2a^^,b^Mean values with no common superscript differ statistically significantly, P < 0.05.

The highest viscosity was found in fresh, thick albumen (P = 0.047). Fresh eggs were characterized by significantly higher albumen viscosity than eggs stored for 7 days, regardless of the air temperature (P = 0.032), using a shear rate of 10 rpm. Taking into account the type of albumen at the shear rate of 10 and 20 rpm, the viscosity of the thick albumen was higher than that of the thin albumen (P < 0.001) (Table 4).

**Table 4:** The viscosity of thick and thin albumen from fresh ducks eggs or after 1 week of storage at different temperatures.

Group^1^	Viscosity (cP)		
	1 rpm	10 rpm	20 rpm
Thin albumen			
*x̅* fresh	9.75^b^	2.56^b^	2.44^bc^
± SD	6.32	0.51	0.42
*x̅* 17 °C	10.30^ab^	2.37^b^	2.32^bc^
± SD	6.57	0.28	0.51
*x̅* 7 °C	8.52^b^	2.62^b^	2.44^c^
± SD	2.92	0.62	0.49
SEM	0.98	0.09	0.08
Thick albumen			
*x̅* fresh	15.60^a^	5.68^a^	3.29^a^
± SD	5.46	3.73	0.57
*x̅* 17 °C	11.19^ab^	3.33^b^	2.72^bc^
± SD	9.28	0.57	0.53
*x̅* 7 °C	9.74^ab^	3.43^b^	2.90^ab^
± SD	1.72	0.77	0.73
SEM	1.20	0.44	0.12
P-value	0.342	0.047	0.383
*x̅* fresh	12.68	4.12^a^	2.86
*x̅* 17 °C	10.75	2.85^b^	2.52
*x̅* 7 °C	9.13	3.03^b^	2.67
P-value	0.177	0.032	0.152
*x̅* Thin albumen	9.52	2.52^b^	2.40^b^
*x̅* Thick albumen	12.18	4.15^a^	2.97^a^
P-value	0.088	< 0.001	< 0.001

^1^Group: Fresh, fresh eggs; 17 °C, eggs stored at 17 °C for 7 days; 7 °C, eggs stored at 7 °C for 7 days.

^2a^^,b^Mean values with no common superscript differ statistically significantly, P < 0.05.

## Discussion

Our research showed a significantly higher egg weight loss at 17 °C (0.94%) compared to 7 °C (0.51%). These results are consistent with research by many authors. Sert et al.^24^ report that chicken eggs stored at 5 °C were characterized by lower weight loss than those stored at 20 °C (regardless of the duration of ultrasound application). A higher decrease in egg weight of Hy-Line Brown hens with increasing temperature was also indicated by Lee et al.^25^ when 2, 12, and 25 °C were used, respectively. It is believed that temperature is a crucial factor in the intensity of this process^26^, which may also explain the lower albumen weight. The weight and percentage of albumen were significantly lower in stored eggs than in fresh ones. Chung and Lee^27^ indicated that hen eggs stored at 25 °C were characterized by significantly lower albumen weight than eggs kept at a lower temperature, corresponding to our research results. The loss of egg weight during storage is primarily due to water's evaporation through the eggshell's pores. In this case, the mechanism of water diffusion between structures also played a key role^28,29^. Higher egg weight loss due to higher temperature and long storage time may also negatively impact the survival of developing embryos during the incubation process^6,30^. Contrary to our research, other authors did not demonstrate the influence of storage temperature on changes in eggshell weight^27,29^.

Egg storage significantly increased the yolk weight and its percentage. Dassidi et al.^7^ showed a significantly higher weight and percentage of yolk in Cherry Valley duck eggs only after 14 days of storage. The air temperature was 12.5 °C, and humidity was 84.59%. Akter et al.^31^ presented similar results after storing chicken eggs at 4 °C and 28–31 °C for 28 days. Lower temperature significantly slowed the increase in the percentage of yolk in the egg (P < 0.005). Water moved from the albumen to the yolk during egg storage, which may explain the increase in the previously mentioned parameters^28^.

The egg yolks stored at 17 °C were characterized by a more intense color on the DSM scale. In addition, higher lightness and yellowness were found compared to the other groups. It corresponds to the results reported by Jin et al.^29^, where a lighter yolk color was found in eggs stored at higher temperatures. Similar to the study reported by Lee et al.^25^, the quality of eggs stored at 2 °C, 12 °C, and 25 °C was compared. It was indicated that temperature is responsible for weakening the vitelline membrane. It was confirmed by research by Chen et al.^32^, in which the influence of time and temperature of egg storage on their quality was analyzed. The cited authors showed the lowest force and energy needed to crack the vitelline membrane in eggs kept at 22 °C, compared to 10 °C and 4 °C. Other authors also indicate similar relationships^33,34^. During egg storage, water is transferred from the albumen to the yolk, which can largely contribute to the lower strength of the vitelline membrane^29^. It could explain the changes in the color of the yolk, particularly its lightening. However, no significant differences in the vitelline membrane strength between the groups were found in our study. Carotenoids, yellow-orange yolk dyes, are susceptible to oxidants, impacting a loss of color intensity^35^. During storage, oxygen penetrates the egg and, as one of the oxidants, could have contributed to increasing the L* value of the yolk, and the higher storage temperature could have accelerated this process.

Storing eggs at a higher temperature resulted in a lower height of thick albumen and a lower HU value than the other groups. Pandian et al.^36^ showed reduced HU values when duck eggs were stored for 4 weeks at 65°F and 75% humidity. A significantly lower HU value in duck eggs was also found at a very early stage of storage—after 3 days^10^. These parameters are considered leading indicators of egg quality, particularly egg freshness^37^. The thinning of the thick albumen fraction intensifies with the length of egg storage. However, this aspect also pays attention to the importance of temperature. The thick albumen's stability is maintained by forming a complex of ovomucin with lysozyme and other proteins in the egg content (ovotransferrin, ovalbumin)^38^. Dam^39^ presented the relationship between the pH value, temperature of egg storage, and the ability to form a lysozyme complex with ovomucin. In duck eggs, it was found that the ability to form connections between these proteins was much lower, regardless of the pH of the environment (from 7.2 to 10.4) at 40 °C compared to 2 °C. Therefore, it can be concluded that this mechanism could have contributed to significantly lower values in the 17 °C group.

Albumen pH (thin and thick) was significantly the highest in the 17 °C group. Similar changes were found in the yolk pH. However, the values differed significantly between the groups (17 °C < 7 °C < fresh). Similar results were presented by Heath^40^ and showed significant changes in the hen's egg albumen pH depending on the storage temperature. Higher temperature (20 °C) increased albumen pH (9.4) compared to eggs stored at 7 °C (9.0) or fresh eggs (8.3). Other authors also confirmed the influence of temperature in albumen pH variations on duck eggs using 28–30 °C and 4 °C^41^. Lengkey et al.^42^ also noticed a significant increase in pH in eggs from ducks during 30 days of storage (from 8.30 to 9.60). Water and carbon dioxide (CO_2_) evaporation through the multiple pores in the eggshell is crucial during egg storage^14,43^.

Mueller^44^ showed a connection between water evaporation and the release of CO_2_ from eggs during storage. The loss of CO_2_ and the penetration of oxygen (O_2_) into the egg interior is responsible for the increase in the pH of the albumen^45^. The temperature is also believed to favor this process significantly^28^. It may explain this parameter's significantly higher value in the albumen from eggs stored at 17 °C in our research. Moreover, as Walsh et al.^30^ pointed out, the storage temperature, the loss of CO_2,_ and changes in albumen pH affect embryo mortality. Contrary to Lee et al.^25^, temperature did not affect pH changes in hen egg yolk. Wang et al.^38^ showed an increase in the pH of the yolk with the extension of egg storage time. Moreover, a temperature of 22 °C affected the increase of the pH of the yolk. The authors also indicated the high importance of temperature in lipase activity, which is responsible for lipid degradation. The higher egg storage temperature resulted in a significantly higher egg albumen and egg yolk pH. In hatching eggs, these changes may accelerate biochemical processes, declining egg biological value through increased embryo mortality.

Lysozyme activity ranged from 7786.67 to 14,980 U/ml in our research. Significantly higher muramidase activity was demonstrated in fresh thin albumen compared to thick albumen. In contrast to our study, Lewko and Gornowicz^46^ indicate a much higher lysozyme activity in the egg albumen of ducks of various genotypes (18,511–31,291 U/ml). Similarly, Yao et al.^47^ reported an activity value of 18,300 U/mg analyzed in salted duck egg albumen. The higher activity of lysozyme in the thin albumen than in the thick albumen was confirmed by other authors investigating the quality of hen eggs^48,49^. According to Fang et al.^50^, a decrease in pH and thinning of thick albumen may contribute to the decline in the antimicrobial capacity of lysozyme. The increase in the pH of the albumen is one of the protective barriers against the penetration of pathogenic microorganisms into the egg content. Our research found lower albumen pH in fresh eggs, with no significantly lower lysozyme activity than eggs stored at 17 °C. No adverse effect of storage temperature may indicate the high defensive capability of the egg contents regardless of the impact of this factor, which may be critical in the further suitability of the eggs for incubation.

Albumen viscosity was significantly higher in fresh eggs than in stored eggs, regardless of the temperature. Therefore, this indicates a higher worth of storage time for quality indicators. Similarly, Kemps et al.^22^ demonstrated a decrease in albumen viscosity from 0.0304 Pa/s to 0.0181 Pa/s after 24 days of storage of hen eggs or Spada et al.^51^ after 14, 28, and 36 days. The results reported by Nangtaodum et al.^52^ confirmed a significant decrease in the viscosity of hen egg albumen after 7 days of storage (from 10.35 mPa/s to 7.24 mPa/s), which corresponds to the results of our study. The ovomucin highly affects the maintenance of the albumen's rheological properties. Its content in the egg significantly decreases during egg storage, and the temperature may be a factor intensifying albumen liquefaction^38^.

Similarly, Lee et al.^25^ indicated that the storage temperature impacts reducing the albumen viscosity. The viscosity between days 10 and 20 decreased from 7.03 to 3.55 units and temperature-related from 7.79 (2 °C) to 0.31 (25 °C). Changes in the rheological properties of egg albumen (viscosity decline) resulted primarily from the liquefaction of thick albumen through biochemical changes. It leads, e.g., to the release of bound molecules by hydrolysis of amino acid chains or the breakdown of the O-glycosidic bond between trisaccharides^53^. Both at 10 rpm and 20 rpm, a significantly higher viscosity of the thick albumen (4.15 cP and 2.97 cP) was found compared to the thin albumen (2.52 cP and 2.40 cP). This relation could have resulted primarily due to the albumen structure, which highly depends on viscosity. The thick albumen is gel-structured, and the thin albumen is liquified^54^. In our research, storage temperature had no significant effect on the viscosity of egg albumen. Significantly higher viscosity was found in fresh eggs compared to stored eggs. Albumen viscosity measured at 10 rpm was 0.18 cP higher in eggs stored at a lower temperature (quantitative). The viscosity of albumen is one of the protective barriers due to the potential limitation of the movement of microorganisms to the developing embryo or blastoderm before incubation. Therefore, it may be critical in obtaining high-quality ducklings and shaping production efficiency^55^.

In conclusion, storage of Cherry Valley duck eggs at 17 °C and 7 °C for 7 days significantly increased egg yolk weight and decreased egg weight and albumen percentage. Higher egg weight loss at 17 °C storage confirmed dynamic water evaporation from the egg, which could also indicate changes in the weight and percentage of albumen and yolk. The share of yolk increased due to the evaporation of water from the albumen. Higher temperatures adversely affected the qualitative characteristics of eggs, such as the height of thick albumen, HU, or the pH of albumen and yolk. Based on the results, it can be concluded that egg storage at a lower temperature significantly reduced physicochemical changes in the morphological structures of eggs, which may determine their higher biological value. On the other hand, the egg storage during 7 days did not negatively affect changes in lysozyme activity and albumen viscosity, which is necessary for the effectiveness of the defense mechanisms of potentially developing embryos. Analyzing selected quality parameters (egg weight loss, HU units, albumen, and yolk pH) indicated a beneficial effect of storage of ducks hatching eggs for 7 days at 7 °C. These results may be necessary in shaping the economics of production and logistics (storing eggs in warehouses and effective hatchability). However, research in this area should be continued, particularly emphasizing hatchability efficiency.

## Material and methods

### Egg collection

In the study, 90 fresh hatching eggs of 52-week-old Cherry Valley ducks (SM3 European Medium) from the parent flock were obtained (Greater Poland Voivodeship, Poland). The eggs were bought commercially. The eggs were collected from breeders on the farm for up to 24 h (farm declaration). Eggs with an average weight of 87.02 g were divided (randomly) into three equal groups. The first group (1-day-old eggs) consisted of fresh eggs. The following two groups were stored under controlled conditions for 7 days (7-day-old eggs). The air temperature remained constant at 17 °C (62.60°F) and 7 °C (44.60°F), respectively, and the air humidity was 70%. Storage conditions were monitored using an electronic thermo-hygrometer (TFA-Dostmann, Wertheim-Reicholzheim, Germany),

### Morphological features and physicochemical composition of eggs

The morphological composition and physicochemical properties of fresh eggs and eggs stored at different temperatures were determined. Eggs were weighed on the first day (fresh ones) and after seven days of storage. (Radwag, Radom, Poland). The length (long axis) and width (short axis) were measured with a caliper. The egg shape index was calculated: 1 $00\times \left(\frac{width}{length}\right)$, as well as the eggshell surface using formula: 4.67 $\times$ W^0.6667^, where W is an egg weight^56^. Eggshell strength (Egg Force Reader, Orka Food Technology Ltd.) and vitelline membrane strength (TA.XT plus C Texturemeter; Stable Micro Systems, Godalming, England) were measured. An apparatus for measuring the strength of the yolk vitelline membrane and a computer program for egg quality analysis (Stable Micro Systems Ltd., UK, Godalming, England) were used. The maximum load of the device was 50 kg. The force per 1 g of eggshell was also calculated ($\frac{eggshell \,strength\, [N]}{eggshell\, weight \,[g]}$).

The weight and percentage of the egg’s albumen, yolk, and eggshell were determined. The eggshells were dried at 105 °C for three hours in a laboratory oven (SUP 10 M). The color of the yolk was analyzed with a colorimeter (Konica Minolta, Tokyo, Japan) on a three-point scale L* (lightness), a* (redness), and b* (yellowness), as well as on a 15 points DSM color scale. After measuring the height of the thick albumen (QCD apparatus from TSS, York, England), the HU was calculated from the formula: 100 lg (H − 1.7 W^0.37^ + 7.57), where H, thick albumen height (mm); W, egg weight (g)^57^.

Yolk, thin, and thick albumen were collected into Falcon tubes (15 ml) using an automatic pipette with a maximum volume of 5000 µl (Sartorius, Goettingen, Germany). The pH of the yolk, thin and thick albumen was measured with a SevenEasy pH meter (Mettler-Toledo AG, Schwerzenbach, Switzerland) using a measuring electrode for liquids. The eggshells were dried in the oven at 100 °C for 3 h, and then eggshell thickness was measured with a micrometer (TSS, York, England)^48,58^.

### Lysozyme activity

For the analysis of the enzymatic activity of lysozyme in egg albumen, a buffer was prepared by adding 20.125 ml of 1 M potassium dihydrogen phosphate solution, 7.375 1 M dipotassium hydrogen phosphate solution, and 550 ml of distilled water to a 1000 cm^3^ beaker. The solution was stirred with a magnetic stirrer (SH-II-4C, Chemland, Poland), and the pH was measured with a SevenEasy pH meter (Mettler-Toledo AG, Schwerzenbach, Switzerland). It was lowered to the appropriate value of 6.25 with 1 M HCl. Previously collected albumen samples from each research group (5 samples each) were thawed, and 1 ml was collected into a 15 ml Falcon tube. A tenfold albumen dilution with buffer was mixed using a vortex mixer (Lab Dancer, IKA, Poland).

Triplicates were prepared by collecting 1 ml of diluted albumen into 1.5 ml Eppendorf and centrifuging (Labnet International, Inc., Sepctrafuge Mini Centrifuge, C1301P-230 V, 6000 rpm, Edison, NJ) for 5 min. In a 250 cm^3^ beaker, a suspension of *Micrococcus lysodeikticus (luteus)* (Merck, Darmstadt, Germany) bacteria was prepared in the buffer, so its absorbance was in the range of 0.600–0.700 nm (λ = 450 nm). The bacterial suspension (2.5 ml) was added to the spectrophotometric cuvette to measure the lysozyme activity, followed by 0.1 ml of the albumen solution. Absorbance was measured with a spectrophotometer (SP830 Plus, Metertech, Taipei, Taiwan) before albumen addition and after 60 s. The blank test consisted of replacing the test sample with the same amount of buffer and testing the decrease in absorbance within 60 s. A lysozyme activity was defined as a decrease in the absorbance of the bacterial suspension by 0.001 nm at a wavelength of 450 nm^58–60^. The following formula was used to calculate lysozyme activity: $\frac{(\mathrm{\Delta \,A}450/{\text{min}}\,Test- \mathrm{\Delta\, A}450/{\text{min}}\,Blank)(df)}{(0.001)(0.1)}$, where df, dilution factor.

### Viscosity

Each group collected 5 thick and thin albumen samples for viscosity analysis. The analyses used a rheometer adapted to small volumes of liquids (Brookfield Ametek, DVNext, LV, Labo Plus, Warsaw, Poland). Albumen (1 ml) was collected into 15 ml Falcon tubes, and a tenfold dilution was made. The samples were thoroughly mixed using a vortex (Lab Dancer, IKA, Poland). Then, from the prepared dilutions, 2 replicates of 1 ml were prepared in 1.5 ml Eppendorf, centrifuged for 5 min (Labnet International, Inc., Sepctrafuge Mini Centrifuge, C1301P-230 V, 6000 rpm, Edison, NJ). Next, 0.5 ml of sample was taken on a rheometer plate and analyzed at three shear rates (1 rpm = 7.50 1/s; 10 rpm = 75.00 1/s; 20 rpm = 150.00 1/s). The obtained results were the average value of all results for 30 s of each analysis^58^.

### Statistical analyzes

Calculations were done using a statistical program (Statistica, Statsoft, 13.0, TIBCO, Cracow, Poland). The data were presented as descriptive statistics, i.e., mean values (*x̅*), standard deviation (± SD), and standard error of the mean (SEM). Statistically significant differences were analyzed by analysis of variance. Statistical verification was performed assuming P < 0.05, using Tukey's test. The statistical model was used for one-way (Y_s_ = µ + C_s_ + e_s_) analysis of variance (ANOVA), where Y_s_, the dependent variable; µ, the overall mean; C_s_, the effect of storage (s: fresh, 7 °C, 17 °C); e_s_, residual error.

### Ethics

The experiment followed the applicable regulations on protecting animals used for scientific or educational purposes. The study concerned the analysis of the physicochemical characteristics of eggs obtained (bought) from a commercial farm of duck breeders. Animals were not used in this research. According to Directive No. 2010/63/EU, the study did not require approval from a Local Ethics Committee. The study is following ARRIVE guidelines.

## Data Availability

The datasets analyzed during the current study are available from the corresponding author upon reasonable request. If there are some questions, the authors remain at your disposal.
